# Human leukocyte antigen class I (A, B) and class II (DRB1) allele and haplotype frequencies in Iranian patients with Buerger's disease

**DOI:** 10.1002/iid3.325

**Published:** 2020-06-21

**Authors:** Fatemeh Dehghani Firouzabadi, Javad Salimi, Aliakbar Amirzargar, Mohammad Dehghani Firouzabadi, Hani Arbabi, Seyyed M. Mousavizadeh, Kambiz Izadpanah

**Affiliations:** ^1^ Department of Vascular Surgery, Sina Hospital Tehran University of Medical Science Tehran Iran; ^2^ Department of Immunology, School of Medicine Tehran University of Medical Sciences Tehran Iran; ^3^ ENT and Head and Neck Research Center and Department, Five Senses Institute, Hazrat Rasoul Akram Hospital Iran University of Medical Sciences Tehran Iran; ^4^ Endocrinology and Metabolism Research Center (EMRC), Vali‐Asr Hospital, School of Medicine Tehran University of Medical Sciences Tehran Iran; ^5^ Project Management Department Tarbiat Modares University Tehran Iran; ^6^ Department of Plastic Surgery, 15 Khordad Educational Hospital Shahid Beheshti University of Medical Sciences Tehran Iran

**Keywords:** Buerger's disease, HLA‐DNA typing, MHC, polymerase chain reaction

## Abstract

**Objective:**

The aim of this study was to investigate the human leukocyte antigen (HLA) class I (HLA‐A and HLA‐B) and II (HLA‐DRB1) allele and haplotype frequencies in a group of Iranian patients with Buerger's disease (BD) in comparison with a normal healthy control group.

**Methods:**

A total of 70 unrelated male patients and 100 healthy controls from Sina Hospital, Tehran, Iran, belonging to the same ethnic background, were enrolled in this case‐control study. HLA‐A, B, and DRB1 typing were performed by polymerase chain reaction with sequence‐specific primers (PCR‐SSP).

**Results:**

The results of this case‐control study showed that the frequency of the HLA‐A*03:01 (odds ratio (OR) = 2.88, *P* value (*Pv*) = .002), HLA‐A*29:01 (OR = 15.31, *Pv* < .001), HLA‐DRB1*04:02 (OR = 3.41, *Pv* < .001), and HLA‐DRB1*16:01 (OR = 8.16, *Pv* < .001) was significantly higher in BD patients compared with healthy controls, whereas the frequency of the HLA‐DRB1*01:01 (OR = 0.03, *Pv* < .001) was significantly lower in BD patients. The most frequent extended haplotypes in our patients were HLA‐A*02:01‐B*55:01‐DRB1*04:03.

**Conclusion:**

This study is the first study evaluating an association between the HLA pattern and BD in the patients with BD from North West and North Iran.

## Graphical abstract

We conducted a case control study to investigate the HLA class I (A and B) and II (DRB1) allele and haplotype frequencies in Iranian patients with Buerger's disease. The study demonstrated that a genetic marker in the HLA region may increase the risk of HLA‐associated susceptibility genes in Iranian patients with Buerger's disease

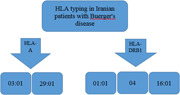

**Abbreviations**
AS: ankylosing spondylitis
BD: Buerger's disease
CI: confidence intervals
MHC: major histocompatibility complex
MS: multiple sclerosis
*Pc*: corrected *P* value
PCR‐SSP: polymerase chain reaction with sequence‐specific primers
*Pv*: *P* values
RA: rheumatoid arthritis
T1D: type 1 diabetes mellitus
TAO: thromboangiitis obliterans

## 1 INTRODUCTION

Thromboangiitis obliterans (TAO) or Buerger's disease (BD) is a segmental occlusive inflammatory condition of arteries and veins, characterized by thrombosis of the affected vessels. It is a non‐atherosclerotic inflammatory disease affecting small and medium‐sized arteries and veins of the upper and lower extremities.^1^ This disease occurs worldwide, but the highest incidence of BD is reported in the Middle and the Far East.^2^ The high incidence of BD in Japan, Sri Lanka (formerly Ceylon), Indonesia, India, Iran, and Mediterranean countries including Turkey, has been reported.^3^, ^4^, ^5^, ^6^, ^7^ The prevalence of the disease among all patients with peripheral arterial disease ranges from values as low as 0.5% to 5.6% in Western Europe, to values as high as 45% to 63% in India, and 16% to 66% in Korea and Japan.^8^ Although the incidence of the disease in Iran cannot be determined exactly due to the lack of a nationwide epidemiological survey, it is widely accepted on the basis of clinical experience that Iran is one of the Middle Eastern countries in which BD frequently occurs.^3^, ^9^

The specific etiology of the disease remained unclear. Secondary etiological factors which have a positive effect on BD disease include age, sex, race, hereditary factor (human leukocyte antigen (HLA) phenotype), autoimmune process, occupation, blood changes (coagulability, anticardiolipin antibodies, homocysteine), and smoking.^10^, ^11^ Epidemiological studies have indicated that autoimmune mechanisms, such as the anti‐collagen types I, III, and IV response^12^ and genetic factors may play a significant role in disease pathogenesis.^13^

HLA allele and haplotype frequency analysis in BD patients revealed an association with the Aw24(A*24), Bw40(B*40), Bw54(B*54), Cw1(C*1), and DR2 antigens and a low frequency of DR9 and DRw52 antigens compared with those observed in normal unaffected Japanese individuals.^14^ In a study by Chen et al,^12^ a positive association with the DPB1*05:01 and DRB1*15:01 was reported, and CD14 genotyping showed that the CD14 TT genotype increased significantly in Japanese patients with BD. Recently, Shapouri‐Moghaddam et al^13^ investigated four HLA‐A subtypes as well as 5 HLA‐B and HLA‐DRB subtypes in patients with BD in Eastern Iranian population in Mashhad, Iran.

As Sina and Imam University hospitals are the largest referral centers in Iran and many patients with BD attend the vascular surgery unit, the aim of the present case‐control study was to investigate the HLA class I (A and B) and II (DRB1) allele and haplotype frequencies in a group of Iranian patients with BD and a normal healthy control group.

## 2 MATERIAL AND METHODS

### 2.1 Study subjects

A total of 70 unrelated patients that had been diagnosed with BD based on Shionoya's criteria^15^ who attended the vascular surgery clinic of Sina Hospital affiliated with Tehran University of Medical Science, were randomly selected. Hundred healthy controls were also randomly selected from blood donors at Iranian blood transfusion organizations. Diagnosis of BD was based on Shionoya's criteria,^15^ which included history of smoking, disease onset before the age of 50, occlusive lesions in the infrapopliteal artery, either upper limb involvement or phlebitis migrans, and the absence of risk factors for atherosclerosis except for smoking. None of the controls had a family history of symptoms related to BD. The patients and controls represented a heterogeneous ethnic population from North West and North Iran, originating from the states of Azerbaijan and Tehran.

### 2.2 Ethical approval

This study protocol was approved by the local ethics committee of the Tehran University of Medical Sciences. The study protocol conformed to the ethical guidelines of the 1975 Declaration of Helsinki as reflected by a priori approval by the institution's Human Research Committee. Written informed consent was obtained from all participants before the study.

### 2.3 HLA typing

DNA was extracted from the 5‐mL venous blood samples taken from each of the participants using a modified salting‐out method. HLA‐A, B, and DRB1 typing was performed by polymerase chain reaction with sequence‐specific primers (PCR‐SSP), following Olerup and Zetterquist's method.^16^ The HLA‐A, B, and DRB primers were supplied by Heidelberg University (Heidelberg, Germany). Polymerase chain reaction (PCR) reactions were carried out in 10 μL volumes and amplification was carried out in a Techne Genius thermal cyclers. Cycling conditions included an initial denaturation step at 94°C for 2 minutes, followed by 10 cycles of denaturation at 94°C for 10 seconds and annealing and extension at 65°C for 60 seconds, then 20 cycles of denaturation at 94°C for 10 seconds, annealing at 61°C for 50 seconds and extension at 72°C for 30 seconds. After amplification, the PCR products were electrophoresed on an agarose gel at 170 V for 15 minutes, and then DNA was visualized on an ultra‐violet (UV) transilluminator.

### 2.4 Statistical analysis

Statistical analysis was performed using GraphPad Prism version 5 (GraphPad Software Inc, 2010, La Jolla, CA). Categorical outcomes were introduced as proportions. A design‐based *χ* ^2^ or Fisher's exact test were run to assess the differences in the frequency and distribution of each allele. The odds ratio and its 95% confidence intervals (CI) were calculated for the association of BD with HLA‐A, B, and DRB alleles. For the multiple analysis performed on each locus, *P* values (*Pv*) were corrected for multiple comparisons and the corrected *P* value (*Pc*) was calculated by dividing the *P* value by the number of alleles in each locus using Bonferroni correction.^17^ A corrected *P* value (*Pc*) of .05 or less was considered to be significant.

## 3 RESULTS

In this matched case‐control study, the patients were in the age group of 22 to 65 years with a mean age of 39.8 years. All patients were male and heavy smokers, with a history of smoking 12 to 38 cigarettes per day (mean of 24 per day) for a period ranging between 8 and 30 years (mean 20 years).

The results of DNA typing analysis for the HLA‐A, B, and DRB alleles of 70 Iranian patients with BD and the control group are shown in Tables 1, 2, 3.

**Table 1:** Frequencies of HLA‐A alleles in BD patients and control subjects

HLA‐A alleles	Patient allele frequencies N = 140	Control allele frequencies N = 200	Odds ratio	95% CI	*P* value	*Pc*
01:01	14 (10.0%)	19 (9.5%)	1.06	0.51‐2.19	1	n.s.
02:01	26 (18.6%)	37 (18.5%)	1.00	0.58‐1.75	1	n.s.
03:01	28 (20.0%)	16 (8.0%)	2.88	1.49‐5.55	.002	Significant
11:01	6 (4.3%)	26 (13.0%)	0.3	0.12‐0.75	.045	n.s.
23:01	4 (2.9%)	8 (4.0%)	0.71	0.21‐2.39	.767	n.s.
24:02	10 (7.1%)	30 (15.0%)	0.44	0.20‐0.92	.027	n.s.
26:01	4 (2.9%)	22 (11.0%)	0.24	0.08‐0.71	.006	n.s.
29:01	10 (7.1%)	1 (0.5%)	15.31	1.94‐121.10	<.001	Significant
30:01	4 (2.9%)	8 (4.0%)	0.71	1.94‐121.10	.767	n.s.
31:01	2 (1.4%)	5 (2.5%)	0.57	0.11‐2.96	.704	n.s.
32:01	14 (10.0%)	11 (5.5%)	1.91	0.84‐4.34	.141	n.s.
33:01	6 (4.3%)	2 (1.0%)	4.43	0.88‐22.30	.069	n.s.
66:01	2 (1.4%)	0 (0.0%)	7.24^a^	0.34‐152	.169	n.s.
68:01	6 (4.3%)	12 (6.0%)	0.71	0.26‐1.92	.625	n.s.
68:02	0 (0.0%)	2 (1.0%)	0.28^a^	0.01‐5.93	.514	n.s.
69:01	4 (2.9%)	1 (0.5%)	5.85	0.65‐52.96	.164	n.s.

Abbreviations: BD, Buerger's disease; HLA, human leukocyte antigen; *Pc*, corrected *P* value; n.s., not significant.

a Odds ratio was calculated by adding 0.5 to each value.

**Table 2:** Frequencies of HLA‐B alleles in BD patients and control subjects

HLA‐B alleles	Patients allele frequencies N = 140	Control allele frequencies N = 180^a^	Odds ratio	95% CI	*P* value	*Pc*
07:02	2 (1.4%)	4 (2.2%)	0.64	0.11‐3.53	.670	n.s.
07:03	0 (0.0%)	2 (1.1%)	0.25^b^	0.01‐5.34	.506	n.s.
07:05	3 (2.1%)	0 (0.0%)	9.18^b^	0.47‐179.50	.083	n.s.
08:01	4 (2.9%)	8 (4.4%)	0.56	0.19‐2.14	.561	n.s.
13:01	2 (1.4%)	8 (4.4%)	0.31	0.06‐1.49	.195	n.s.
14:02	2 (1.4%)	3 (1.7%)	0.86	0.14‐5.19	1	n.s.
15:01	6 (4.3%)	3 (1.7%)	2.64	0.65‐10.76	.187	n.s.
15:03	4 (2.9%)	0 (0.0%)	11.90^b^	0.63‐223.10	.036	n.s.
18:01	8 (5.7%)	6 (3.3%)	1.76	0.59‐5.19	.410	n.s.
35:01	18 (12.9%)	27 (15.0%)	0.84	0.44‐1.59	.629	n.s.
37:01	2 (1.4%)	5 (2.8%)	0.51	0.01‐2.655	.474	n.s.
38:01	8 (5.7%)	15 (8.3%)	0.67	0.27‐1.62	.393	n.s.
39:01	0 (0.0%)	4 (2.2%)	0.14^b^	0.01‐2.62	.134	n.s.
40:01	0 (0.0%)	2 (1.1%)	0.25^b^	0.01‐5.34	.506	n.s.
40:02	4 (2.9%)	2 (1.1%)	2.62	0.47‐14.51	.41	n.s.
41:01	6 (4.3%)	4 (2.2%)	1.97	0.54‐7.12	.343	n.s.
44:02	6 (4.3%)	5 (2.8%)	1.57	0.47‐5.25	.543	n.s.
44:03	10 (7.1%)	8 (4.4%)	1.65	0.63‐4.31	.334	n.s.
49:01	6 (4.3%)	1 (0.6%)	8.02	0.95‐67.40	.046	n.s.
50:01	6 (4.3%)	16 (8.9%)	0.46	0.17‐1.21	.123	n.s.
51:01	14 (10.0%)	29 (16.1%)	0.58	0.29‐1.14	.137	n.s.
52:01	12 (8.6%)	9 (5.0%)	1.78	0.73‐4.36	.256	n.s.
55:01	12 (8.6%)	4 (2.2%)	4.13	1.30‐13.09	.017	n.s.
57:01	0 (0.0%)	8 (4.4%)	0.07^b^	0.004‐1.26	.010	n.s.

Abbreviations: BD, Buerger's disease; HLA, human leukocyte antigen; *Pc*, corrected *P* value; n.s., not significant.

a For HLA‐B locus, 90 healthy controls were randomly selected from blood donors at Iranian blood transfusion organizations that were enrolled in this study.

b Odds ratio was calculated by adding 0.5 to each value.

**Table 3:** Frequencies of HLA‐DRB1 alleles in BD patients and control subjects

HLA‐DRB alleles	Patients allele frequencies	Normal control alleles frequencies	Odds ratio	95% CI	*P* value	*Pc*
	N = 140	N = 200				
01:01	0 (0.0%)	18 (9.0%)	0.03^a^	0.002‐0.59	<.001	Significant
03:01	14 (10.0%)	18 (9.0%)	1.12	0.54‐2.34	.851	n.s.
04	32 (22.9%)	16 (8.0%)	3.41	1.79‐6.50	<.001	Significant
07	10 (7.1%)	26 (13.0%)	0.51	0.24‐1.10	.107	n.s.
08:01	0 (0.00%)	8 (4.0%)	0.08^a^	0.004‐1.41	.023	n.s.
09:01	0 (0.00%)	2 (1.0%)	0.28^a^	0.01‐5.93	.514	n.s.
10:01	2 (1.4%)	6 (3.0%)	0.46	0.09‐2.36	.478	n.s.
11:01	27 (19.3%)	38 (19.0%)	1.02	0.59‐1.76	1	n.s.
11:02	2 (1.4%)	10 (5.0%)	0.27	0.06‐1.28	.132	n.s.
12	1 (0.7%)	0 (0.0%)	4.31^a^	0.17‐106.70	.412	n.s.
13:01	8 (5.7%)	12 (6.0%)	0.95	0.38‐2.39	1	n.s.
13:02	0 (0.0%)	6 (3.0%)	0.10^a^	0.01‐1.91	.045	n.s.
13:03	0 (0.0%)	2 (1.0%)	0.28^a^	0.01‐5.93	.514	n.s.
14:01	2 (1.4%)	8 (4.0%)	0.34	0.07‐1.66	.206	n.s.
15:01	22 (15.7%)	26 (13.0%)	1.24	0.67‐2.31	.528	n.s.
16:01	20 (14.3%)	4 (2.0%)	8.16	2.72‐24.47	<.001	Significant

Abbreviations: BD, Buerger's disease; HLA, human leukocyte antigen; *Pc*, corrected *P* value; n.s., not significant.

a Odds ratio was calculated by adding 0.5 to each value.

In the HLA‐A locus, the frequency of the A*03:01 (20% vs 8%, *Pv* = .002, *Pc* = significant) and A*29:01 (7.1% vs 0.5%, *Pv* < .001, *Pc *= significant) was significantly higher in patients compared with the healthy controls, whereas the frequency of the A*11:01 and A*26:01 was lower in patients (4.3% vs 13%, *Pv* = .044% and 2.9% vs 11%, *Pv* = .006, respectively) but did not reach statistical significance after correction of the *P* value (Table 1).

In the HLA‐B locus, the frequency of the B*55:01 (8.6% vs 2.2%, *Pv* = .017), B*15:03 (2.9% vs 0%, *Pv* = .035), and B*49:01 (4.3% vs 0.6%, *Pv* = 0.046) was increased in patients compared with healthy controls but did not reach statistical significance after correction of the *P* value. The frequency of the B*57:01 (0% vs 4.4%, *Pv* = 0.010) was decreased in the patient group compared with the control group but this increase was not statistically significant after correction of the *P* value (Table 2).

In the HLA‐DRB1 locus, the DRB1*04 and DRB1*16 were significantly increased (22.8% vs 8%, *Pv* < .001, *Pc* = significant and 14.3% vs 2%, *Pv* < .001, *Pc* = significant, respectively), whereas the DRB1*01:01 was significantly reduced in patients compared with the healthy controls (0.0% vs 9%, *Pv* < .001, *Pc* = significant) (Table 3).

The frequency of the DRB1*08:01 and DRB1*13:02 (0% vs 4%, *Pv* = .023% and 0% vs 3%, *Pv* = .045, respectively) were increased in the control group compared with the patient group but this was not significant after correction of the *P* value. The most frequent DRB1*04 subtype in our patients was the DRB1*04:02 (from 16 patients that were DRB1*04, 12 were DRB1*04:02, and 4 were DRB1*04:03).

## 4 DISCUSSION

The extremely high levels of polymorphism and heterozygosity within the major histocompatibility complex (MHC) genomic region provide the immune system with a selective advantage against the diversity and variability of pathogens. However, the high rate of mutation within MHC is accompanied by the added risk of generating autoimmune diseases and other genetic disorders. Several hundred autoimmune and infectious diseases have been associated with the MHC genetic region, including type 1 diabetes mellitus (T1D), rheumatoid arthritis (RA), multiple sclerosis (MS), and ankylosing spondylitis (AS), however, the exact molecular mechanisms are unknown.^18^ Like other autoimmune diseases, BD is a multi‐factorial disease with predisposing genetic backgrounds and environmental factors. The association between HLA alleles and haplotypes and BD has been reported by various investigators in different populations in endemic regions.^4^, ^12^, ^19^, ^20^, ^21^ Vijayakumar et al^22^ found that patients diagnosed with BD have different forms in HLA haplotype and heterogeneity which is common between them. Our study revealed the higher frequency of the A*03:01, A*29:01, DRB1*04, and DRB1*16:01 and the lower the frequency of the DRB1*01:01 in patients compared with healthy controls.

HLA analysis of BD patients in India^4^ demonstrated a positive association with the B40 and DR2 (DRB1*15:01, *15:02, and *16:02). Although Iranian and some Indian populations originate from Indo‐European Arian ancestors, the frequency of the B*4001 and 4002 did not differ between Iranian BD patients and healthy controls but the DR2, specifically DRB1*16, increased significantly in Iranian patients with BD, similarly to Indian BD patients. These differences in allele frequencies in Indian and Iranian populations may be partially attributable to the typing methodology, but also reflect ethnicity differences between these populations.

HLA analysis of Japanese patients with BD^12^, ^19^, ^23^ revealed a positive association with the B*54:01, DRB1*15:01, DRB1*04:05, and DPB1*05:01 and a negative association with the DRB1*13:02 and DRB1*04:01. Our findings in Iranian patients with BD confirmed earlier reports on the association of BD with DRB1*04 in Japanese patients. The most frequent DRB1*04 subtypes in our patients were DRB1*04:02 and DRB1*04:03. Similarly, to the Japanese study, we found that the frequency of the DRB1*13:02 was higher in healthy controls compared with BD patients, but did not reach statistical significance after correction of the *P* value (3% vs 0%, *Pv* = 0.044). Other alleles were not found to differ significantly in our population. A recent study in an Eastern Iranian sample population, Shapouri‐Moghaddam et al^13^ showed that the HLA‐associated gene in patients with BD was associated with four HLA‐A subtypes (ie, A*03, A*24, A*31, and A*11), five HLA‐B subtypes (ie, B*27, B*15, B*07, B*51, and B*44), and five HLA‐DRB1 subtypes (ie, DRB1*16, DRB1*04, DRB1*14, DRB1*03, and DRB1*15). Moreover, they found that A*25, A*66, DRB1*08, DRB1*10, and DRB1*12 was associated with an increased risk of BD. Similar to that study, our study with larger sample size of patients with BD from North West and North Iran found that the frequencies of A*03, DRB1*04, and DRB1*16 were increased in the patients. In contrast to that study that showed HLA‐B*08, B*45, B*46, and B*53 had a protective role against BD, our study found that DRB1*10:01 was decreased in the Buerger's patients. Our findings also showed that A*02:01‐B*55:01‐DRB1*04:03 was the most frequent extended haplotype in the patients. The limitation of this study is its small sample size, as these kind of studies require larger sample sizes to achieve an adequate statistical power.

## 5 CONCLUSION

In conclusion, our findings like other studies suggest that a genetic marker in the HLA region along with some environmental such as smoking and immunological factors may increase the risk of HLA‐associated susceptibility genes in Buerger's patients. Further studies with a larger sample size are needed to confirm these results and establish the role of HLA alleles and haplotypes in BD association.

## CONFLICT OF INTERESTS

The authors declare that there are no conflict of interests.

## ETHICS STATEMENT

All procedures performed in studies involving human participants were in accordance with the ethical standards of the institutional and/or national research committee and with the 1964 Helsinki declaration and its later amendments or comparable ethical standards.

## Data Availability

All data generated or analyzed during this study are included in this submitted article.

## References

[1] Olin JW , Young JR , Graor RA , Ruschhaupt WF , Bartholomew JR . The changing clinical spectrum of thromboangiitis obliterans (Buerger's disease). Circulation. 1990;82(5 suppl):Iv3‐Iv8. 2225420

[2] Lie JT . Thromboangiitis obliterans (Buerger's disease) revisited. Pathol Annu. 1988;23(Pt 2):257‐291. 3060814

[3] Salimi J , Tavakkoli H , Salimzadeh A , Ghadimi H , Habibi G , Masoumi AA . Clinical characteristics of Buerger's disease in Iran. J Coll Physicians Surg Pak. 2008;18(8):502‐505. 18798588

[4] Jaini R , Mandal S , Khazanchi RK , Mehra NK . Immunogenetic analysis of Buerger's disease in India. Int J Cardiol. 1998;66(suppl 1):S283‐S285. 9951831 10.1016/s0167-5273(98)00180-6

[5] Inada K , Hayashi M , Okatani T . Chronic occlusive arterial disease of lower extremity in Japan: with special reference to Buerger's disease. JAMA Surg. 1964;88(3):454‐460. 10.1001/archsurg.1964.01310210128022 14088277

[6] Kradjian R , Bowles LT , Edwards WS . Peripheral arterial disease in Ceylon. Surgery. 1971;69(4):523‐525. 5553916

[7] Hill GL , Smith AH . Buerger's disease in Indonesia: clinical course and prognostic factors. J Chronic Dis. 1974;27(4):205‐216. 4843253 10.1016/0021-9681(74)90046-0

[8] Arkkila PET . Thromboangiitis obliterans (Buerger's disease). Orphanet J Rare Dis. 2006;1(1):14. 16722538 10.1186/1750-1172-1-14 PMC1523324

[9] Tavakoli H , Rezaii J , Esfandiari K , Salimi J , Rashidi A . Buerger's disease: a 10‐year experience in Tehran, Iran. Clin Rheumatol. 2008;27(3):369‐371. 18172574 10.1007/s10067-007-0784-x

[10] Dellalibera‐Joviliano R , Joviliano EE , Silva JS , Evora PR . Activation of cytokines corroborate with development of inflammation and autoimmunity in thromboangiitis obliterans patients. Clin Exp Immunol. 2012;170(1):28‐35. 22943198 10.1111/j.1365-2249.2012.04624.x PMC3444714

[11] Malecki R , Kluz J , Przezdziecka‐Dolyk J , Adamiec R . The pathogenesis and diagnosis of thromboangiitis obliterans: is it still a mystery? Adv Clin Exp Med. 2015;24(6):1085‐1097. 26771983 10.17219/acem/33322

[12] Chen Z , Takahashi M , Naruse T , et al. Synergistic contribution of CD14 and HLA loci in the susceptibility to Buerger disease. Hum Genet. 2007;122(3‐4):367‐372. 17653770 10.1007/s00439-007-0408-1

[13] Shapouri‐Moghaddam A , Mohammadi M , Rahimi HM , et al. The association of HLA‐A, B and DRB1 with Buerger's disease. Rep Biochem Mol Biol. 2019;8(2):153‐160. 31832439 PMC6844607

[14] Numano F , Sasazuki T , Koyama T , et al. HLA in Buerger's disease. Exp Clin Immunogenet. 1986;3(4):195‐200. 3274054

[15] Shionoya S . Diagnostic criteria of Buerger's disease. Int J Cardiol. 1998;66(suppl 1):S243‐S245. Discussion S247. 9951826 10.1016/s0167-5273(98)00175-2

[16] Olerup O , Zetterquist H . HLA‐DR typing by PCR amplification with sequence‐specific primers (PCR‐SSP) in 2 hours: an alternative to serological DR typing in clinical practice including donor‐recipient matching in cadaveric transplantation. Tissue Antigens. 1992;39(5):225‐235. 1357775 10.1111/j.1399-0039.1992.tb01940.x

[17] Svejgaard A , Ryder LP . HLA and disease associations: detecting the strongest association. Tissue Antigens. 1994;43(1):18‐27. 8023317 10.1111/j.1399-0039.1994.tb02291.x

[18] Shiina T , Hosomichi K , Inoko H , Kulski JK . The HLA genomic loci map: expression, interaction, diversity and disease. J Hum Genet. 2009;54(1):15‐39. 19158813 10.1038/jhg.2008.5

[19] Kimura A , Kobayashi Y , Takahashi M , et al. MICA gene polymorphism in Takayasu's arteritis and Buerger's disease. Int J Cardiol. 1998;66(suppl 1):S107‐S113. Discussion S115. 9951809 10.1016/s0167-5273(98)00157-0

[20] Mehra NK , Jaini R . Immunogenetics of peripheral arteriopathies. Clin Hemorheol Microcirc. 2000;23(2‐4):225‐232. 11321444

[21] Papa M , Bass A , Adar R , et al. Autoimmune mechanisms in thromboangiitis obliterans (Buerger's disease): the role of tobacco antigen and the major histocompatibility complex. Surgery. 1992;111(5):527‐531. 1598672

[22] Vijayakumar A , Tiwari R , Prabhuswamy VK . Thromboangiitis obliterans (Buerger's disease)—current practices. Int J Inflammat. 2013;2013:156905. 10.1155/2013/156905 PMC3786473 24102033

[23] Aerbajinai W , Tsuchiya T , Kimura A , Yasukochi Y , Numano F . HLA class II DNA typing in Buerger's disease. Int J Cardiol. 1996;54(suppl):S197‐S202. 9119524 10.1016/s0167-5273(96)88790-0

